# Haplotype-resolved genome of ‘Pinot Noir’ reveals DNA polymorphisms and allele-specific expression shaping terroir adaptation

**DOI:** 10.1186/s43897-025-00226-3

**Published:** 2026-06-04

**Authors:** Lingfei Shangguan, Yanhua Ren, Xuxian Xuan, Shaonan Li, Dan Pei, Xiaobei Chen, Rui Zhang, Desheng Mou, Xin Wang, Weiping Chen, Meilong Xu, Xiangpeng Leng, Le Guan, Jiali Chen, Shuo Wang, Huihui Fan, Haider Muhammad Salman, Jinggui Fang

**Affiliations:** 1 College of Horticulture, Nanjing Agricultural University, Nanjing, Jiangsu Province 210095 China; 2 Fruit Crop Genetic Improvement and Seedling Propagation Engineering Research Center of Jiangsu Province, Nanjing, Jiangsu Province 210095 China; 3 Shandong Academy of Forestry, Jinan, Shandong Province 250014 China; 4 Institute of Economic Forest, Wuwei Academy of Forestry Science, Wuwei, Gansu Province 733004 China; 5 Institute of Horticulture, Ningxia Academy of Agriculture and Forestry Sciences, Ningxia, Ningxia Hui Autonomous Region 750002 China; 6 College of Horticulture, Qingdao Agricultural University, Qingdao, Shandong Province 266109 China; 7 College of Life Science, Northeast Forestry University, Harbin, Heilongjiang Province 150040 China; 8 Department of Horticulture, Ghazi University, Dera Ghazi Khan, Punjab 32200 Pakistan

Grapevine (*Vitis* spp.) is a globally important crop used for wine, raisins, juice, and fresh fruit. Its genome, the first sequenced from a fruit crop, provided insights into plant evolution and trait mechanisms, with over 40 varieties now sequenced (Wang et al. 2024b). However, high heterozygosity, up to 13% divergence between homologous chromosomes, and widespread clonal propagation complicate genome assembly (Zhou et al. 2019). Most existing assemblies are consensus sequences representing a single haplotype, masking allelic diversity, while the inbred line PN40024 inadequately represents commercial cultivars (Characterization TFIPCfGG 2007). For the historic wine variety ‘Pinot Noir’ (PN), recent pan-genomic studies have produced haplotype-resolved assemblies but lack in-depth analyses of structural variations (SVs), allelic expression, and terroir-associated adaptations (Li et al. 2024).

We validated our assembly approach by re-sequencing PN40024, generating PN4_2022 genome, which showed a 370-fold improvement in contig N50 (24.47 Mb) and anchored 139.02 Mb of previously unplaced sequences (Figs. S2–S4; Tables S1–S7). Applying the same approach to PN produced a consensus assembly (529.39 Mb) and two haplotypes (PN_hap1: 521.70 Mb; PN_hap2: 512.86 Mb). All assemblies showed high completeness and contiguity (contig N50 > 20 Mb, anchoring > 95%, BUSCO > 98%) (Fig. S5a, S6, Tables S1, S8–S9). Synteny analysis covered ~ 72% of the chromosomes, revealing haplotype-specific variation in gene density (13.61–19.73 kb/gene) and functional enrichments, notably in secondary metabolism on Chr06/10 (Fig. 1a; Figs. S5b, S7–S8; Tables S10–S13). The consensus assembly masked ~ 1,000 haplotype-specific genes in non-aligned regions, most of which were enriched in metabolic functions (Tables S14–S18).

**Fig. 1:**
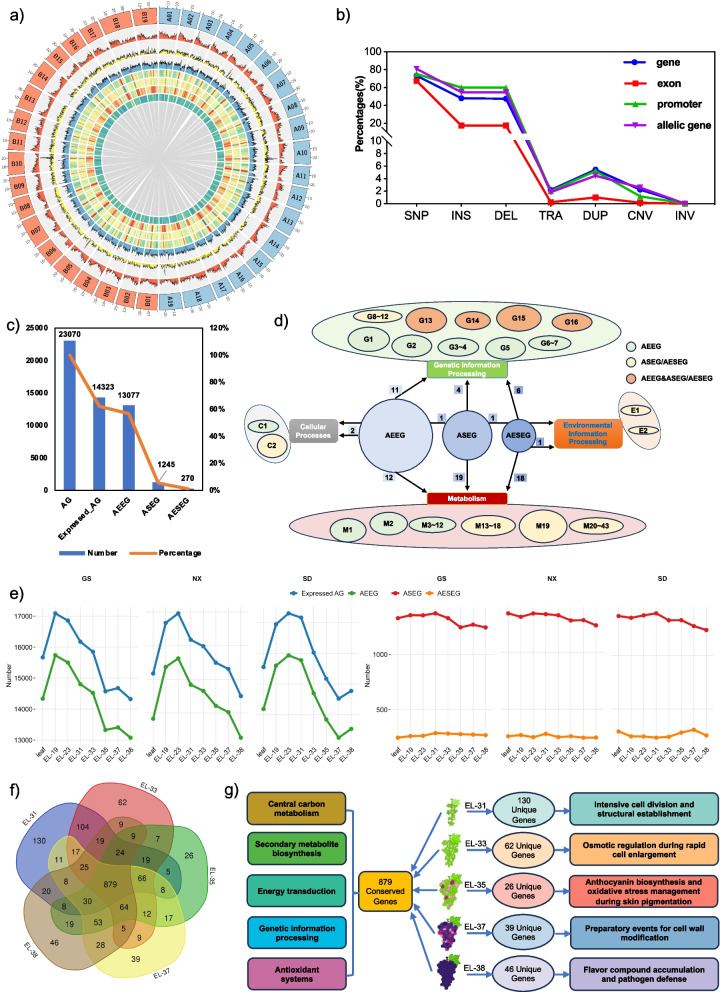
The genomic architecture, allelic variation, and functional roles of ‘PN’. **a** The circular tracks represent the following elements (from outer to inner): (1) schematic representation of Hap1 (A) and Hap2 (B) chromosome sequences; (2) gene density; (3) SNP density; (4) SSR loci density; (5) density of *Gypsy* elements; (6) density of *Copia* elements; (7) density of DNA transposons; (8) GC content; and (9) synteny relationships; **b** Percentages of different types of polymorphisms across various gene structures; **c** Numbers and proportions of different types of allelic genes in G_PN_EL-38. AG, allelic genes; AEEG, allele-equivalent expressed genes; ASEG, allele-specific expressed genes; AESEG, allele-extremely specific expressed genes; **d** KEGG pathway analysis of AEEG, ASEG, and AESEG in G_PN_EL-38. Genetic Information Processing: G1, Ubiquitin-mediated proteolysis; G2, Spliceosome; G3, SNARE interactions in vesicular transport; G4, Ribosome biogenesis in eukaryotes; G5, Protein processing in the endoplasmic reticulum; G6, Protein export; G7, Nucleocytoplasmic transport; G8, Nucleotide excision repair; G9, Mismatch repair; G10, Homologous recombination; G11, DNA replication; G12, Aminoacyl-tRNA biosynthesis; G13, RNA degradation; G14, Proteasome; G15, mRNA surveillance pathway; G16, Basal transcription factors. Metabolism: M1, Carbon metabolism; M2, Biosynthesis of amino acids; M3, Glycine, serine and threonine metabolism; M4, Citrate cycle (TCA cycle); M5, Inositol phosphate metabolism; M6, 2-Oxocarboxylic acid metabolism; M7, Arginine biosynthesis; M8, Pentose phosphate pathway; M9, One-carbon pool by folate; M10, Carbon fixation in photosynthetic organisms; M11, Glycosylphosphatidylinositol (GPI)-anchor biosynthesis; M12, Glyoxylate and dicarboxylate metabolism; M13, Fructose and mannose metabolism; M14, Glycerolipid metabolism; M15, Galactose metabolism; M16, N-Glycan biosynthesis; M17, Glutathione metabolism; M18, Various types of N-glycan biosynthesis; M19, Metabolic pathways; M20, Pentose and glucuronate interconversions; M21, Ascorbate and aldarate metabolism; M22, Biosynthesis of secondary metabolites; M23, Glycolysis/Gluconeogenesis; M24, Linoleic acid metabolism; M25, Porphyrin metabolism; M26, Folate biosynthesis; M27, Neomycin, kanamycin and gentamicin biosynthesis; M28, Purine metabolism; M29, Steroid biosynthesis; M30, Terpenoid backbone biosynthesis; M31, Starch and sucrose metabolism; M32, Amino sugar and nucleotide sugar metabolism; M33, Fatty acid degradation; M34, Betalain biosynthesis; M35, Limonene and pinene degradation; M36, Lysine degradation; M37, Biosynthesis of various plant secondary metabolites; M38, Histidine metabolism; M39, Pantothenate and CoA biosynthesis; M40, Pyruvate metabolism; M41, Tryptophan metabolism; M42, Flavonoid biosynthesis; M43, Phenylpropanoid biosynthesis. Cellular Processes: C1, Autophagy – other; C2, Endocytosis. Environmental Information Processing: E1, ABC transporters; E2, MAPK signaling pathway – plant; **e** Numbers of different types of allelic genes in GS, NX, and SD. AG, AEEG, ASEG, and AESEG are as defined above; **f **Venn diagram of ASEGs among different fruit developmental stages; **g** Functional annotation of conserved and unique ASEGs across fruit development stages

In total, we annotated 63,044 genes, including 26,235 two-copy genes (23,070 allelic and 3,165 non-allelic), 10,574 single-copy genes (SCGs), and 36,809 cultivar-wide genes (CWGs) (Tables S1, S19, Fig. S9a). LTRs, primarily *Copia*/*Gypsy* elements, were the dominant repetitive sequences (~ 37.5%) (Fig. 1a, Tables S8, S20–S21).

Our approach revealed extensive polymorphisms between PN homologs, identifying 3,579,066 SNPs, 335,561 insertions (INSs), 337,312 deletions (DELs), 2,564 translocations (TRAs), 8,818 duplications (DUPs), 1,748 copy number variations (CNVs), and 63 inversions (INVs), totaling 4,265,132 variants. SNPs/INSs/DELs predominated (99.69%), with 86% of variations < 10 bp, while TRAs/DUPs/CNVs/INVs were rare (0.31%) and over 94% were > 1 kb (Fig. S9b–h; Table S11). Homologous sequence identity ranged from 52.67 to 88.96%, with length differences up to 3.99 Mb (Fig. S5a; Tables S11–S12). Variants were predominantly non-centromeric, as 94.94–100% of SNPs/INSs/DELs/CNVs/INVs occurred between homologous regions, while 69.85–82.41% of DUPs/TRAs were heterologous (Table S22). SNP density varied from 123,820 to 262,544 per chromosome (104–245 bp/SNP; Fig. S10, Table S11). These polymorphism patterns mirror other grapevine genomes (Wang et al. 2024a) and diverse plant species (Han et al. 2023; Zhang et al. 2021), reflecting a conserved trend of high-frequency small variants like SNPs and InDels (≤ 10 bp).

Most SNPs/INSs/DELs were located in intergenic (~ 70%) or intronic (25–26%) regions, with 2.58–5.56% in exonic, and 17–23% in promoter regions (Fig. S11a–11b; Table S23). Within coding regions, we identified 68,842 synonymous and 87,976 nonsynonymous SNPs (*Ts*/*Tv* = 1.8734; 65.19% transitions). High-impact variants (0.045–0.333%) affected 1,597–2,555 genes, which were enriched in categories related to binding, DNA metabolism, signaling, and phosphorylation, while 8,315 variants disrupted 5,799 genes (Tables S24–S25). Structural variants (TRAs/DUPs/CNVs/INVs) extensively overlapped genic regions (45.54–49.69%), exons (9.55–30.05%), and promoters (22.14–35.66%) (Figs. S9h, S11c; Table S23). These SVs impacted a substantial proportion (45.54–69.84%) of genes (1–377), many associated with growth and stress responses (Fig. 1b; Tables S26–S29). Variants occurring in allelic genes exerted stronger disrupted effects than those on non-allelic genes (Fig. 1b; Tables S23, S29–S30).

Allelic gene pairs with small variants showed higher sequence divergence but lower *Ka*/*Ks* ratios, suggesting stronger purifying selection (Figs. S11d–e). Furthermore, we identified 298,572 simple sequence repeats (SSRs; 1,542–1,999 bp/locus), of which > 80% were polymorphic. These were predominantly composed of mono/di/tri-nucleotide motifs (> 97%), mainly AT/TA and AAT/ATT repeats, and were distributed across genic (25%), promoter (29%), and exonic (1.94%) regions (Fig. S12; Tables S31–S33).

We performed RNA-seq analysis across tissues (flowers, leaves, berries) from the GS region and mapped the reads to the haplotypes. In mature berries (EL-38 stage), 62.08% of allelic pairs (14,323) were transcriptionally active. These were classified as: 1,245 allele-specific expressed genes (ASEGs), 270 allele-extremely specific expressed genes (AESEGs; expressed exclusively from one allele), and the remainder as allele-equivalently expressed genes (AEEGs) (Fig. 1c; Table S34). This allelic expression bias is common in heterozygous genomes (Zhang et al. 2021; Cheng et al. 2021). AEEGs were enriched in core biological pathways, including carbon metabolism and protein synthesis, supporting cellular and developmental stability. Conversely, ASEGs/AESEGs were enriched in secondary metabolic and stress-response pathways, reflecting their contribution to developmental plasticity and environmental adaptation (Fig. 1d; Fig. S13; Table S35). GO enrichment confirmed that AEEGs maintain transcriptional stability, ASEGs contribute to cell expansion and signaling, and AESEGs participate in RNA splicing and fruit softening (Figs. S14–S16; Tables S36–S37). ASEGs/AESEGs exhibited higher *Ka*/*Ks* ratios (0.64/0.63 *vs*. 0.46), indicating positive selection pressure (Table S38). This allelic expression bias was associated with DNA polymorphisms, as ASEGs/AESEGs contained a higher frequency of high-impact variants. Such regulatory variations likely drive expression preference, promoting phenotypic diversity and adaptive evolution (Han et al. 2023; Li et al. 2024).

Across berry development (EL-31 to EL-38), the number of expressed allelic genes (AGs) ranged from 14,323 to 16,175 (AEEGs: 13,077–14,802; ASEGs: 1,246–1,373; AESEGs: 270–289), peaking at the pea-size stage (EL-31) and reaching the lowest at the harvest-ripe stage (EL-38) (Fig. 1e; Table S34). A core set of 879 ASEGs across different fruit stages was enriched in pathways related to carbon/secondary metabolism, energy production, antioxidant activity, cell expansion, sugar accumulation, pigmentation, and cell wall remodeling (Fig. 1f–g; Fig. S17; Table S39). Stage-specific ASEGs included genes involved in cell division at EL-31 and anthocyanin biosynthesis/stress response at EL-35 (Fig. 1g; Table S40). Cross-tissue comparisons showed that flowers expressed the highest number of AGs, while fruits exhibited the most AESEGs (270; Fig. 1e; Table S34). The 854 ASEGs shared across tissues were enriched in core metabolic processes, genetic information processing, stress adaptation, and post-translational regulation (Figs. S17, S18a–b; Table S41). Conversely, tissue-specific ASEGs were functionally linked to organ-specific activities: cell wall biosynthesis/modification (fruit), reproductive processes (flowers), and protein phosphorylation/complex assembly/modification (leaves) (Fig. S18b; Table S42).

Across NX/SD berries, AGs ranged from 14,502 to 17,307 (AEEGs: 13,251–15,934; ASEGs: 1,236–1,392; AESEGs: 233–307). Overall, 68.74% of allelic genes were expressed across all samples (63.02% AEEGs, 5.72% ASEGs, and 1.13% AESEGs) (Fig. 1e; Table S34). The *Ka*/*Ks* ratios again indicated stronger positive selection on ASEGs (0.65–0.67) and AESEGs (0.59–0.65) compared with AEEGs (0.46–0.47) (Fig. S18c; Table S38). Functional analyses supported this conserved pattern: AEEGs regulated core biological pathways, while ASEGs/AESEGs were enriched in sugar-lipid homeostasis, stress responses, and secondary metabolite biosynthesis (Figs. S19–S25; Tables S35–S37). Additionally, ASEGs showed distinct region-specific expression, highlighting their potential role in environmental plasticity. For example, ASEGs from the arid GS region were enriched in N-glycan and β-glucan metabolic pathways associated with drought adaptation, while those from the NX region were enriched in lipid biosynthesis, potentially contributing to aroma accumulation (Figs. S25–S27; Tables S39–S42). Collectively, ASEGs mediate a balance between transcriptional stability and adaptive plasticity, consistent with patterns observed in bamboo climate variants (Hou et al. 2024) and grape polymorphisms (Marrano et al. 2018).

DNA polymorphisms directly modulated allele-specific expression. ASEGs/AESEGs had a higher density of SNPs/INSs/DELs than AEEGs (Fig. S28a; Table S43). Promoter DELs frequently biased allelic expression (*e.g*., three DELs in *LOB* gene induced extreme expression levels; Fig. S28b; Table S44). SVs also contributed: INV6868 (9.79 kb, Chr02) disrupted an allelic pair, converting it into six SCGs, while TRANS6939 (8.41 kb, Chr01) transformed three allelic pairs into SCGs (Figs. S28c–e). Terroir conditions further influenced fruit quality, as SD-region grapes exhibited higher organic acid contents than GS/NX grapes (Fig. S29). Gene expression correspondingly varied: *Malate synthase* was upregulated at the SD EL-38, whereas *LDH* was downregulated compared with NX/GS, possibly reflecting climate-driven transcriptional regulation (Table S45). Together, these findings highlight that environmental variability, in conjunction with DNA polymorphisms, modulates allelic expression to shape plant adaptability and phenotypic traits (Gil and Ulitsky 2020; Xu et al. 2025).

We generated a high-quality, haplotype-resolved ‘PN’ genome, revealing extensive inter-haplotype divergence and dynamic allelic expression. Our findings indicated that small-scale variants primarily contribute to metabolic stability, whereas structural variants and regulatory polymorphisms promote biased allelic expression, driving environmental adaptation and phenotypic diversity. This work provides a robust genomic framework for elucidating the molecular basis of grapevine diversity and terroir-influenced fruit quality.

## Supplementary Information

Supplementary Material 1. Material and Methods, with all the supplementary documents were available on Supplementary Materials 1 and 2.

Supplementary Material 2: Table S1. Summary of genome assembly and annotation of *Vitis vinifera* ‘Pinot Noir’ and ‘PN40024’. Table S2. The GO annotation of the genes in PN4_2007, PN4_2022 and PN_2022. Table S3. The brief information of the consensus sequence of ‘PN40024’ genomes (PN4_2022 and PN4_8 × _2007). Table S4. The alignment between PN4_8x_2007 random sequences and PN4_2022 chromosome sequences. Table S5. The information of relocated PN_8x_2007 genes. Table S6. The inversions and translocations between the chromosomes of PN4_8x_2007 and PN4_2022. Table S7. The inversions and translocations between the chromosomes of PN4_12x_2007 and PN4_2022. Table S8. The repetitive sequence of hap1, hap2, and consensus genomes of ‘PN’. Table S9. The GO annotation of the genes in PN4_2007, PN4_2022 and PN_2022. Table S10. The structural variation and sequence variation between PN_hap1 and PN_hap2. Table S11. The structure variation distribution in each chromosome between hap1 and hap2 of ‘PN. Table S12. The sequence similarity between PN_hap1 and PN_hap2. Table S13. The top 25 GO and KEGG enrichment analysis of each chromosome. Table S14. The syntenic and not aligned regions comparison among PN_hap1, PN_hap2, and PN_consensus. Table S15. The details of genes located on the not aligned regions of PN_hap1 and PN_hap2. Table S16. The GO annotation of the genes located on the not aligned regions of PN_hap1 and PN_hap2. Table S17. The KEGG pathway annotation of the genes located on the not aligned regions of PN_hap1 and PN_hap2. Table S18. The anthocyanin biosynthesis related genes, *STS*, and *TPS* genes in PN_hap1, PN_hap2, and PN_consensus. Table S19. The cultivar whole genes information in PN. Table S20. The LTR/*Copia* distribution on PN_hap1 and PN_hap2. Table S21. The LTR/*Gypsy* distribution on PN_hap1 and PN_hap2. Table S22. The DNA polymorphism distribution between HoC and HeC. Table S23. The distribution information of different polymorphisms on different gene structures. Table S24. SNP, INS and DEL number of effects by impact. Table S25. GO annotation of high-effect SNP, INS and DEL-related genes. Table S26. The GO annotation of gene with polymorphism located on gene regions. Table S27. The GO annotation of gene with polymorphism located on exon regions. Table S28. The GO annotation of gene with polymorphism located on promoter regions. Table S29. The number and percentage of genes with polymorphism located on different regions. Table S29. The GO annotation of gene with polymorphism located on allelic genes. Table S31. The SSR distribution in each chromosome between hap1 and hap2 of ‘PN. Table S32. The SSR motifs analysis between PN_hap1 and PN_hap2. Table S33. The distribution information of SSRs on different gene structures. Table S34. The number and their proportions of expressed AG, AEEG, ASEG, and AESEG within AG. Table S35. The KEGG annotation of AEEGs, ASEGs, and AESEGs in different samples. Table S36 The GO annotation (Biological Process) of AEEGs, ASEGs, and AESEGs in different samples. Table S37. The GO annotation (Cellular Component) of AEEGs, ASEGs, and AESEGs in different samples. Table S38. The *Ka/Ks* value of AEEGs, ASEGs, and AESEGs in different samples. Table S39. The KEGG annotation of common genes among different fruit development stages. Table S40. The GO annotation count (level 3) of unique genes among different fruit development stages. Table S41. The KEGG annotation of common genes among different organs. Table S42. The GO annotation (Biological Process) of unique genes among different organs. Table S43. The SNP/INS/DEL high effected genes distribution in AEE, ASE, AESE shared genes in three regions (GS, SD, NX). Table S44. The details of genes located in DNA polymorphisms. Table S45. The specific organic acid biosynthesis related genes at different fruit developmental stages in different regions.

## Data Availability

All the genome sequences and annotation files were stored in figshare database (https://figshare.com/s/e4100b36377f6e3a8bb1), and RNA-seq data of ‘PN’ has been submitted to China National Center for Bioinformation (CNCB) database with accession ID CRA018239.
