# Supplementary material for: Haplotype-resolved genome of ‘Pinot Noir’ reveals DNA polymorphisms and allele-specific expression shaping terroir adaptation
Source: Mol Hortic. 2026 Jun 4;6:41. doi: 10.1186/s43897-025-00226-3 (PMC13235053; doi:10.1186/s43897-025-00226-3)
Supplement: Supplementary file 1 — Supplementary Material 1. Material and Methods, with all the supplementary documents were available on Supplementary Materials 1 and 2. [file 43897_2025_226_MOESM1_ESM.docx]

**Haplotype-resolved genome of ‘Pinot Noir’ reveals DNA polymorphisms and allele-specific expression shaping terroir adaptation**

Lingfei Shangguan^ab#^, Yanhua Ren^abc#^, Xuxian Xuan^ab^, Shaonan Li^ab^, Dan Pei^ab^, Xiaobei Chen^ab^, Rui Zhang^ab^, Desheng Mou^d^, Xin Wang^d^, Weiping Chen^e^, Meilong Xu^e^, Xiangpeng Leng^f^, Le Guan^g^, Jiali Chen^ab^, Shuo Wang^ab^, Huihui Fan^ab^, Haider Muhammad Salman^h^, Jinggui Fang^ab*^

^a^ College of Horticulture, Nanjing Agricultural University, Nanjing, Jiangsu Province, 210095, China

^b^ Fruit Crop Genetic Improvement and Seedling Propagation Engineering Research Center of Jiangsu Province, Nanjing, Jiangsu Province, 210095, China

^c^ Shandong Academy of Forestry, Jinan, Shandong Province, 250014, China

^d^ Institute of Economic Forest, Wuwei Academy of Forestry Science, Wuwei, Gansu Province, 733004, China

^e^ Institute of Horticulture, Ningxia Academy of Agriculture and Forestry Sciences, Ningxia, Ningxia Hui Autonomous Region, 750002, China

^f^ College of Horticulture, Qingdao Agricultural University, Qingdao, Shandong Province, 266109, China

^g^ College of Life Science, Northeast Forestry University, Harbin, Heilongjiang Province, 150040, China

^h^ Department of Horticulture, Ghazi University, Dera Ghazi Khan, 32200, Pakistan

^#^ These authors equal the contribution.

Corresponding author: Jinggui Fang, fanggg@njau.edu.cn

**Materials and Methods**

**Plant materials**

Four-years-old grapevine (cultivars ‘Pinot Noir’ and ‘PN40024’, abbreviated as ‘PN’ and ‘PN4’) were planted in Baima Teaching and Research Base of Nanjing Agricultural University, Jiangsu Province, China. Young leaves were collected for DNA sequencing, while the mixed sample of leaves, roots, stems, and fruits were collected for RNA sequencing. To investigate gene expression dynamics across different developmental stages and growing regions, additional ‘PN’ samples, including leaves, flowers, and fruits at different developmental stages, were collected from three major wine grape-producing regions in China: Wuwei Academy of Forestry Sciences (Gansu Province, abbreviated as GS or G), Ningxia Academy of Agriculture and Forestry Sciences (eastern foothills of Helan Mountain, Ningxia Hui Autonomous Region, abbreviated as NX or N), and Chateau JunDing (Weihai, Shandong Province, abbreviated as SD or S). The growth stages of the grapevines were determined according to the E-L system (Coombe 1995).

**Genome survey and** **evaluation using next-generation sequencing (NGS) technology**

Firstly, the DNA of ‘PN’ and ‘PN4’ were extracted and randomly fragmented into ~ 250 bp segments using a Covaris ultrasonic processor, respectively. The sequencing library was then prepared and sequenced on the MGI-2000 platform (BGI-Shenzhen, China). Secondly, raw sequencing reads underwent quality control using SOAPnuke (v1.6.5) (Chen et al. 2018), where low-quality reads, adapter sequences, and PCR duplicates were filtered out to generate clean reads. Lastly, the clean reads were subsequently used for genome survey analysis, including the estimation of genome size, heterozygosity, and repeat content using K-mer analysis and the GenomeScope model (Vurture et al. 2017).

**Genome sequencing and assembly using the combined sequencing strategies**

The genome sequences of ‘PN’ and ‘PN4’ were sequenced by PacBio HiFi reads and Hi-C technologies. The sequencing process consisted of two main steps: (i) PacBio HiFi sequencing: A total of 15 μg of genomic DNA was used to construct a SMRTbell 15k library following the standard protocol. The genomic DNA was sheared to the desired fragment size and sequenced on a PacBio Sequel II instrument using Sequencing Primer V2 and Sequel II Binding Kit 2.0 at GrandOmics. (ii) Hi-C sequencing: Hi-C libraries were constructed at BGI-Shenzhen Institute. Genomic DNA was extracted from young leaves of ‘PN’ and ‘PN4’ and digested with MboI, following the standard Hi-C library preparation protocol. The Hi-C libraries were sequenced on the MGI-2000 platform, generating approximately 60 Gb of 150-bp paired-end reads.

For genome assembly, we employed smrtlink, SOAPnuke, and HiFiasm. First, raw sequencing reads from HiFi and Hi-C were filtered using smrtlink (v8.0) and SOAPnuke (v1.65) to remove low-quality sequences. The filtering parameters were set as follows: ‘ccs –minPasses 3 –min-rq 0.99’ for smrtlink and ‘-n 0.01 -l 20 -q 0.1 -i -Q 2 -G -M 2 -A 0.5 -d’ for SOAPnuke. Second, genome assembly was performed using HiFiasm with the following command: ‘-i <input_reads> -t 32 -o’. The clean reads from the filtering step were used for genome assembly.

For validation, the ‘PN4’ assembly was used to generate a consensus sequence and was compared with previously published ‘PN4’ genome versions (PN4_8×_2007 and PN4_12×_2007). The final assembly of the ‘PN’ genome (PN_2022) was completed following the workflow outlined in **Supplementary Fig. S1**.

**Annotation of ‘PN’ and ‘PN4’ haploid genomes**

**Repeat annotation**. A combined strategy of homology alignment and *de novo* prediction was applied to identify the whole genome repeats. Tandem Repeats were identified using TRF (Tandem Repeats Finder, http://tandem.bu.edu/trf/trf.html) via *ab initio* prediction. Homology-based repeat identification was performed using the Repbase database (http://www.girinst.org/repbase) and RepeatMasker (http://www.repeatmasker.org/) with its in-house scripts (RepeatProteinMask) under default parameters to extracted repeat regions (Jurka et al. 2005; Tarailo‐Graovac and Chen 2009). Additionally, *ab initio* repeat identification was conducted using LTR_FINDER (http://tlife.fudan.edu.cn/ltr_finder/), RepeatScout (http://www.repeatmasker.org/), RepeatModeler (http://www.repeatmasker.org/RepeatModeler.html) under default parameters. All identified repeat sequences longer than 100 bp with gaps (N) less than 5% were compiled into a raw transposable element (TE) library (Xu and Wang 2007; Price et al. 2005). To improve repeat annotation, a custom repeat library was created by combining Repbase with the de novo TE library, which was processed with UCLUST to generate a non-redundant dataset. This custom library was then used for DNA-level repeat identification with RepeatMasker.

**Gene Structure Annotation**. Structural gene annotation was performed using a combination of ab initio prediction, homology-based prediction, and RNA-Seq-assisted prediction: i). Ab initio-prediction: Gene models were predicted using Augustus (v3.2.3), Geneid (v1.4), Genescan (v1.0), GlimmerHMM (v3.04), and SNAP (2013-11-29) within an automated gene prediction pipeline (Parra et al. 2000; Stanke et al. 2006; Stanke and Waack 2003; Majoros et al. 2004); ii). Homology-based prediction: Protein sequences from homologous species were retrieved from the Ensembl Plants database (<https://plants.ensembl.org/index.html>), Washington State University Bioinformatics database (<https://www.bioinfo.wsu.edu/>), and UC Davis Viticulture & Ecology database (http://169.237.73.197/Chardonnay04/). These sequences were aligned to the genome using TBLASTN (v2.2.26; E-value ≤ 1e−5), and matching protein sequences were further aligned to the homologous genome regions for precise spliced alignments using GeneWise (v2.4.1), which was employed to predict gene structures (Altschul et al. 1997; Birney et al. 2004); iii). RNA-seq-assisted prediction: Transcriptome assemblies were generated using Trinity (v2.1.1) (Trapnell et al. 2010). RNA-Seq reads from different tissues were mapped to the genome using HISAT (v2.0.4) with the following parameters: hisat2 -p 8 --sensitive --no-discordant --no-mixed -I 1 -X 1000 --max-intronlen 1000000 -x, allowing for the identification of exon regions and splice junctions (Kim et al. 2015). The resulting alignments were subsequently processed with StringTie (v1.3.3) using the command “stringtie --merge gtf.list -o rna.stringtie.gtf.tmp”, enabling genome-guided transcript assembly and refinement of gene models (Pertea et al. 2015). A final non-redundant reference gene set was obtained by integrating predictions from all three approaches using EvidenceModeler (EVM, v1.1.1). PASA (Program to Assemble Spliced Alignment) was used to refine gene models by incorporating terminal exon support, while masked transposable elements were included as input for gene prediction (Haas et al. 2008). Genes of particular interest were subjected to additional manual curation by relevant experts.

**Functional Annotation**. Gene functions were assigned according to the best match by aligning the protein sequences to the Swiss-Prot using blastp (with a threshold of E-value ≤ 1e−5). The motifs and functional domains were annotated using InterProScan (v5.31), which incorporated multiple publicly available databases, including ProDom, PRINTS, Pfam, SMRT, PANTHER and PROSITE (Mulder and Apweiler 2007). Gene Ontology (GO) terms were assigned based on corresponding InterPro entries (Ashburner et al. 2000). Protein functions were further predicted by transferring annotations from the closest BLAST hit (E-value <10^-5^) in the Swiss-Prot database and best DIAMOND (v0.8.22) or BLAST hit (E-value <10^-5^) in the NR database (Bairoch and Apweiler 2000). Additionally, KEGG pathway mapping was performed to identify relevant metabolic and regulatory pathways for each gene (Kanehisa and Goto 2000).

**Structural variation identification and classification**

To identify structural variations (SVs), the haploid genome sequences were aligned using MUMmer (v4.0) with the parameters: nucmer --maxmatch -c 500 -b 500 -l 100 -t 20 (Marçais et al. 2018). Following alignment, SVs were detected and visualized using SyRI with default settings (Goel et al. 2019). The identified SVs were then classified manually based on the SyRI annotation results to ensure accurate categorization.

**RNA-seq**

The ‘PN’ samples of leaves, flowers, and fruits at different development stages were collected from three vineyards (GS, NX, SD) for RNA-sequencing, with each sample replicated three times. Total RNA was extracted using the CTAB method and used for cDNA library construction. RNA-seq was performed on the MGI-2000 platform (BGI-Shenzhen), and raw reads were processed to obtain high-quality clean reads using FastQC and Trimmomatic (Bolger et al. 2014). The clean reads were then independently mapped to the ‘PN’ haplotype genomes (hap1 and hap2) using STAR (Dobin et al. 2013). Gene expression levels were quantified using FeatureCounts to obtain read counts, and TPM values were calculated using edgeR (Liao et al. 2014; Robinson et al. 2010).

**Allele identification and expression analysis**

Alleles between the two haplotypes were identified using the AlleleFinder pipeline (https://github.com/sc-zhang/AlleleFinder), following previously described methods (Wang et al. 2021; Zhang et al. 2018; Misra et al. 2023; Gylemo et al. 2024). Briefly, MCScanX was used to detect syntenic blocks between haplotypes, and genes within these blocks sharing >70% sequence similarity were designated as allelic pairs (Wang et al. 2012). For allele expression analysis, genes were filtered using the following criteria: i) genes were considered expressed if they had a TPM ≥ 1 and read count ≥ 10 in at least one sample; ii) allele-specific expression (ASE) genes were identified using DESeq2 with the thresholds P < 0.05, adjusted P < 0.05, and log₂(fold change) > 1 (Love et al. 2014). Based on expression patterns, allele pairs were categorized into three groups: biallelically expressed genes (also known as Allele Equivalent Expressed Genes, AEEG), which are expressed at similar levels between the two haplotypes; Allele-Specific Expressed Genes (ASEG), which exhibit significantly differential expression between haplotypes; and Allele Extremely Specific Expressed Genes (AESEG), a subset of ASEGs where one allele has TPM < 1 while the other has TPM > 1, indicating monoallelic expression exclusive to either hap1 or hap2. GO and KEGG enrichment analyses, as well as *Ka*/*Ks* ratio calculations, were performed using TBtools (Chen et al. 2020).

**FISH probe design and analysis**

Single-copy oligonucleotide probes were designed following Han et al. (Han et al. 2015). To eliminate repetitive sequences, RepeatMasker (http://www.repeatmasker.org) was applied to the PN4_12×_2007 genome. Subsequently, the genome was fragmented into 48-nt oligonucleotides with a step size of 5 nt using Chorus (https://chorus.readthedocs.io/en/master/) (Zhang et al. 2021). BLAST (Kent 2002) was used to align the generated oligos against the PN40024 reference genome, filtering out sequences with similarity >75% and anchoring multiple locations. The final probe sequences are provided in **Supplemental Document 2** (Pei et al. 2024). Specific probes corresponding to targeted chromosomal regions were selected from the probe database and visualized using Python and R.

For cytological analysis, root tips were collected from cuttings and tissue culture seedlings. Approximately 1.5 cm of root tip was excised and pretreated in a 0.2 μmol/L amiprophos-methyl (APM) solution (dissolved in acetone) for 2 h at 25°C. The APM solution was then discarded, and the root tips were rinsed 2–3 times with distilled water before being transferred to a 1.5 mL centrifuge tube. Samples were treated with nitrous oxide (0.8–1.2 MP) for 30 min, followed by fixation in 90% acetic acid at 4°C for 7 min. The acetic acid was removed, and the root tips were stored in 70% ethanol at -20°C. Chromosome preparation and fluorescence in situ hybridization (FISH) were performed as described by Doležel et al. (Doležel et al. 1992) and Liu et al. (Liu et al. 2020).

**Determination of fruit quality indicators**

Fruit quality traits, including five-berry weight, transverse diameter, and longitudinal diameter, were measured using a digital caliper and precision balance. Total soluble sugars, organic acids, total anthocyanins, and total phenolic content were quantified using the phenol-sulfuric acid method, acid-base titration, pH-differential spectrophotometry, and Folin-Ciocalteu method, respectively (Yang et al. 2004; Lee et al. 2005).

**Fig. S1 The workflow of the ‘Pinot Noir’ genome assembly using NGS, Pacbio sequencing (HiFi), Hi-C technologies.**

**Fig. S2 The sequence and gene comparison between PN4_8×_2007 and PN4_2022 genomes**. (a) The gene proportion in two genomes; (b) CDS average and total lengths comparison between PN4_8×_2007 and PN4_2022 genomes; (c) The chromosome length; (d) the percentage of anchored sequences in two genomes; (e) Synteny regions between PN4_8×_2007 and PN4_2022 consensus chromosome sequences. The synteny regions were linked using different color lines between two genomes; (f) relocated chromosome length and gene number.

**Fig. S3 The syntenic analysis and structural variation among PN4_8×_2007, PN4_2022, and PN4_12×_2007 chromosomes**.

**Fig. S4 The distribution of gene, *copia*, *gypsy*, syntenic and variation regions in PN4_2022 chromosomes compare to PN4_8×_2007 chromosomes.**

**Fig. S5 The genome assembly and annotation of ‘Point Noir’ (PN_2022).** (a) The chromosome length; (b). Gene, SNP, SSR distribution on Chr02.

**Fig. S6 The FISH results of ‘PN’ and ‘PN40024’.** (a) The fluorescence in situ hybridization (FISH) results of ‘PN’ and ‘PN40024’; (b) The FISH probe sketch.

**Fig. S7 The syntenic and structural variation analysis between PN_hap1 (PN_h1) and PN_hap2 (PN_h2) chromosomes**.

**Fig. S8 The syntenic and structural variation analysis among PN_hap1 (PN_h1), PN_consensus (PN_con), and PN_hap2 (PN_h2) chromosomes**.

**Fig. S9 The gene copy (a) and polymorphisms distribution between PN_hap1 and PN_hap2.** The proportion of different polymorphisms (b), and the size distribution of INS (c), DEL (d), TRA (e), DUP (f), INV (g), CNV (h).

**Fig. S10 The distribution of different polymorphisms, gene, and repeat sequences in different chromosomes.**

**Fig. S11 The polymorphism distribution between PN_2022.** (a) SNP, INS and DEL distribution in different gene structures; (b) The proportion of different lengths of SNP, INS, and DEL; (c) TRA, DUP, CNV, and INV distribution in different gene structures; (d) The sequence identity between contained or without SNP/INS/DEL-allelic genes; (e) The Ka/Ks value between contained or without SNP/INS/DEL-allelic genes.


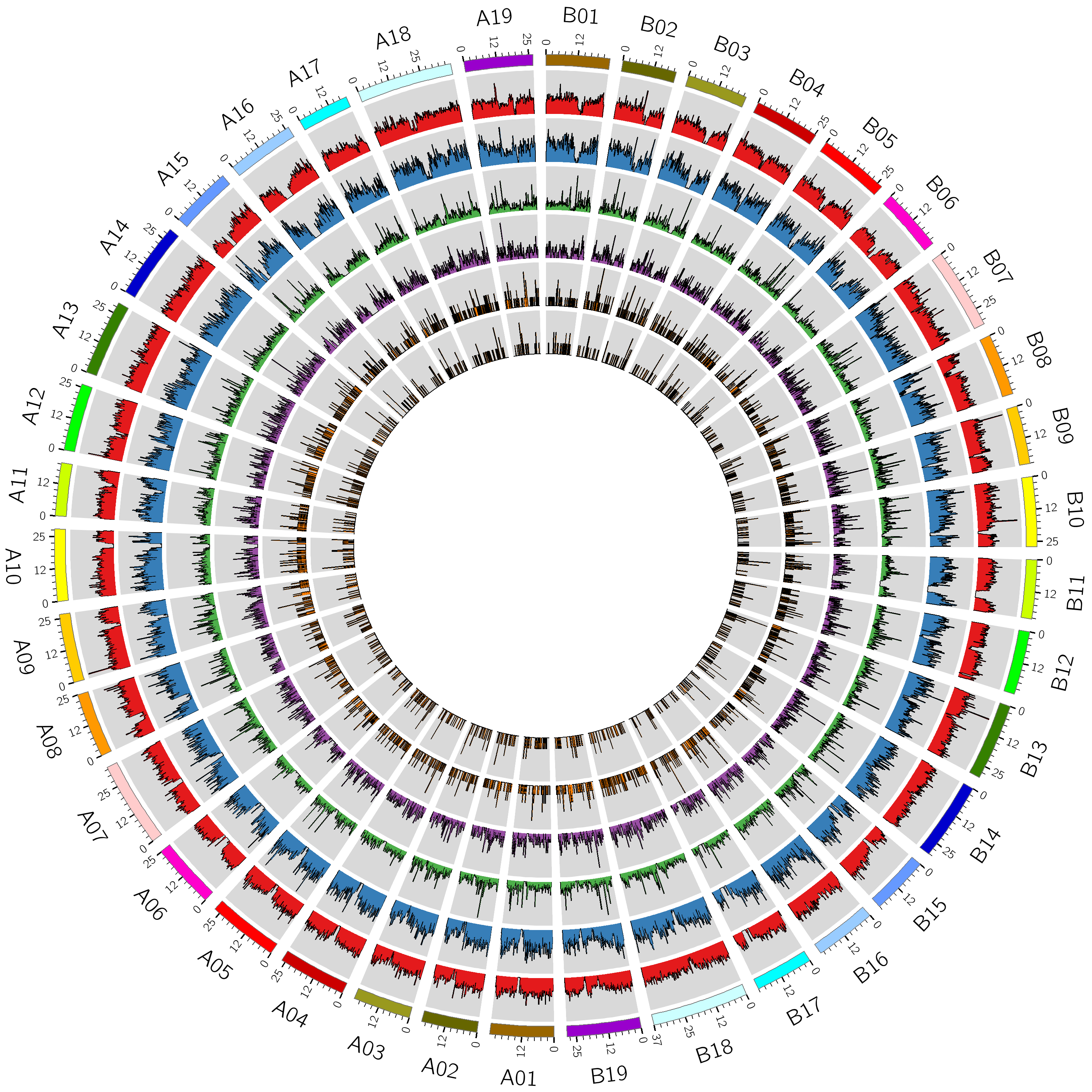


**Fig. S12 The SSR distribution between PN_hap1 and PN_hap2**. The tracks represent the following elements (from outer to inner): (1) schematic representation of Hap1 (A), Hap2 (B) chromosome sequences. (2) Mononucleotide repeats. (3) Dinucleotide repeats. (4) Trinucleotide repeats. (5) Tetranucleotide repeat. (6) Pentanucleotide repeats. (7) Hexanucleotide repeats.

**Fig. S13 The KEGG pathway analysis of AEEG (a), ASEG (b) and AESEG (c) in G_PN_EL-38.**

**Fig. S14 The characteristics of allelic genes during different developmental stages of grapevine in Gansu region.** (a) Top 10 GO biological process (BP) terms for AEEG, ASEG and AESEG in G_PN_EL-38; (b) Top 10 GO cellular component (CC) terms for AEEG, ASEG and AESEG in G_PN_EL-38.

**Fig. S15 The GO annotation (Cellular Component) of AEEG (a), ASEG (b), AESEGs (c) in G_PN_EL-38.**

**Fig. S16 The GO annotation (Biological Process) of AEEG (a), ASEG (b), AESEGs (c) in G_PN_EL-38.**

**Fig. S17 The KEGG enrichment analysis of shared ASEGs among different fruit developmental stages (a) or different organs (b) in Gansu.**

**Fig. S18 The characteristics of allelic genes in different fruit stages or organs**. (a) The venn diagram of ASEGs among different organ; (b) The annotation of conserved and unique ASEGs among different organs; (c) The *Ka*/*Ks* value of AEEG, ASEG, AESEG in different regions.

**Fig. S19 The KEGG pathway analysis of AEEG (a), ASEG (b) and AESEG (c) in N_PN_EL-38.**

**Fig. S20 The GO annotation (Cellular Component) of AEEG (a), ASEG (b), AESEGs (c) in N_PN_EL-38.**

**Fig. S21 The GO annotation (Biological Process) of AEEG (a), ASEG (b), AESEGs (c) in N_PN_EL-38.**

**Fig. S22 The KEGG pathway analysis of AEEG (a), ASEG (b) and AESEG (c) in S_PN_EL-38.**

**Fig. S23 The GO annotation (Cellular Component) of AEEG (a), ASEG (b), AESEGs (c) in S_PN_EL-38.**

**Fig. S24 The GO annotation (Biological Process) of AEEG (a), ASEG (b), AESEGs (c) in S_PN_EL-38.**

**Fig. S25 The annotation of AEEGs, ASEGs, and AESEGs among different regions.**

**Fig. S26 The KEGG enrichment analysis of shared ASEGs among different fruit developmental stages (a) or different organs (b) in Ningxia.**

**Fig. S27 The KEGG enrichment analysis of shared ASEGs among different fruit developmental stages (a) or different organs (b) in Shandong.**

**Fig. S28 The impact of DNA polymorphisms on different allelic genes**. (a). The proportion of highly effected genes of different DNA polymorphisms in the total genes; (b). The DNA polymorphism effect on allelic gene imbalance expression; (c-e). The DNA polymorphism effect on allelic gene formation.

**Fig. S29 Phenotypic (a) and physiological parameters (b) of PN fruits in different regions.**

**References**

Altschul SF, Madden TL, Schäffer AA, Zhang J, Zhang Z, Miller W, Lipman DJ (1997) Gapped BLAST and PSI-BLAST: a new generation of protein database search programs. Nucleic Acids Research 25 (17):3389-3402

Ashburner M, Ball CA, Blake JA, Botstein D, Butler H, Cherry JM, Davis AP, Dolinski K, Dwight SS, Eppig JT (2000) Gene ontology: tool for the unification of biology. Nature Genetics 25 (1):25-29

Bairoch A, Apweiler R (2000) The SWISS-PROT protein sequence database and its supplement TrEMBL in 2000. Nucleic Acids Research 28 (1):45-48

Birney E, Clamp M, Durbin R (2004) GeneWise and genomewise. Genome Research 14 (5):988-995

Bolger AM, Lohse M, Usadel B (2014) Trimmomatic: a flexible trimmer for Illumina sequence data. Bioinformatics 30 (15):2114-2120

Chen C, Chen H, Zhang Y, Thomas HR, Frank MH, He Y, Xia R (2020) TBtools: an integrative toolkit developed for interactive analyses of big biological data. Molecular Plant 13 (8):1194-1202

Chen Y, Chen Y, Shi C, Huang Z, Zhang Y, Li S, Li Y, Ye J, Yu C, Li Z (2018) SOAPnuke: a MapReduce acceleration-supported software for integrated quality control and preprocessing of high-throughput sequencing data. Gigascience 7 (1):gix120

Coombe BG (1995) Growth stages of the grapevine: adoption of a system for identifying grapevine growth stages. Australian Journal of Grape and Wine Research 1 (2):104-110

Dobin A, Davis CA, Schlesinger F, Drenkow J, Zaleski C, Jha S, Batut P, Chaisson M, Gingeras TR (2013) STAR: ultrafast universal RNA-seq aligner. Bioinformatics 29 (1):15-21

Doležel J, Číhalíková J, Lucretti S (1992) A high-yield procedure for isolation of metaphase chromosomes from root tips of *Vicia faba* L. Planta 188:93-98

Goel M, Sun H, Jiao W-B, Schneeberger K (2019) SyRI: finding genomic rearrangements and local sequence differences from whole-genome assemblies. Genome Biology 20:1-13

Gylemo B, Bensberg M, Hennings V, Lundqvist C, Camponeschi A, Goldmann D, Zhang H, Selimović-Pašić A, Lentini A, Ekwall O (2024) A landscape of X-inactivation during human T cell development. Nature Communications 15 (1):1-14

Haas BJ, Salzberg SL, Zhu W, Pertea M, Allen JE, Orvis J, White O, Buell CR, Wortman JR (2008) Automated eukaryotic gene structure annotation using EVidenceModeler and the program to assemble spliced alignments. Genome Biology 9 (1):R7

Han Y, Zhang T, Thammapichai P, Weng Y, Jiang J (2015) Chromosome-specific painting in *Cucumis* species using bulked oligonucleotides. Genetics 200 (3):771-779

Jurka J, Kapitonov VV, Pavlicek A, Klonowski P, Kohany O, Walichiewicz J (2005) Repbase Update, a database of eukaryotic repetitive elements. Cytogenetic and Genome Research 110 (1-4):462-467

Kanehisa M, Goto S (2000) KEGG: kyoto encyclopedia of genes and genomes. Nucleic Acids Research 28 (1):27-30

Kent WJ (2002) BLAT—the BLAST-like alignment tool. Genome Research 12 (4):656-664

Kim D, Langmead B, Salzberg SL (2015) HISAT: a fast spliced aligner with low memory requirements. Nature Methods 12 (4):357-360

Lee J, Durst RW, Wrolstad RE, Kupina CETGMHJHHKSKD, JD SMSMBMTPFRASGTUW (2005) Determination of total monomeric anthocyanin pigment content of fruit juices, beverages, natural colorants, and wines by the pH differential method: collaborative study. Journal of AOAC international 88 (5):1269-1278

Liao Y, Smyth GK, Shi W (2014) featureCounts: an efficient general purpose program for assigning sequence reads to genomic features. Bioinformatics 30 (7):923-930

Liu X, Sun S, Wu Y, Zhou Y, Gu S, Yu H, Yi C, Gu M, Jiang J, Liu B (2020) Dual‐color oligo‐FISH can reveal chromosomal variations and evolution in *Oryza* species. The Plant Journal 101 (1):112-121

Love MI, Huber W, Anders S (2014) Moderated estimation of fold change and dispersion for RNA-seq data with DESeq2. Genome Biology 15:1-21

Majoros WH, Pertea M, Salzberg SL (2004) TigrScan and GlimmerHMM: two open source ab initio eukaryotic gene-finders. Bioinformatics 20 (16):2878-2879

Marçais G, Delcher AL, Phillippy AM, Coston R, Salzberg SL, Zimin A (2018) MUMmer4: A fast and versatile genome alignment system. PLoS Computational Biology 14 (1):e1005944

Misra CS, Sousa AG, Barros PM, Kermanov A, Becker JD (2023) Cell-type-specific alternative splicing in the Arabidopsis germline. Plant Physiology 192 (1):85-101

Mulder N, Apweiler R (2007) Interpro and interproscan. In: Comparative genomics. Springer, pp 59-70

Parra G, Blanco E, Guigó R (2000) Geneid in drosophila. Genome Research 10 (4):511-515

Pei D, Yu X, Fu W, Ma X, Fang J (2024) The evolution and formation of centromeric repeats analysis in *Vitis vinifera*. Planta 259 (5):99

Pertea M, Pertea GM, Antonescu CM, Chang T-C, Mendell JT, Salzberg SL (2015) StringTie enables improved reconstruction of a transcriptome from RNA-seq reads. Nature Biotechnology 33 (3):290

Price AL, Jones NC, Pevzner PA (2005) *De novo* identification of repeat families in large genomes. Bioinformatics 21 (suppl_1):i351-i358

Robinson MD, McCarthy DJ, Smyth GK (2010) edgeR: a Bioconductor package for differential expression analysis of digital gene expression data. Bioinformatics 26 (1):139-140

Stanke M, Schöffmann O, Morgenstern B, Waack S (2006) Gene prediction in eukaryotes with a generalized hidden Markov model that uses hints from external sources. BMC Bioinformatics 7 (1):62

Stanke M, Waack S (2003) Gene prediction with a hidden Markov model and a new intron submodel. Bioinformatics 19 (suppl_2):ii215-ii225

Tarailo‐Graovac M, Chen N (2009) Using RepeatMasker to identify repetitive elements in genomic sequences. Current Protocols in Bioinformatics 25 (1):4.10. 11-14.10. 14

Trapnell C, Williams BA, Pertea G, Mortazavi A, Kwan G, Van Baren MJ, Salzberg SL, Wold BJ, Pachter L (2010) Transcript assembly and quantification by RNA-Seq reveals unannotated transcripts and isoform switching during cell differentiation. Nature Biotechnology 28 (5):511

Vurture GW, Sedlazeck FJ, Nattestad M, Underwood CJ, Fang H, Gurtowski J, Schatz MC (2017) GenomeScope: fast reference-free genome profiling from short reads. Bioinformatics 33 (14):2202-2204

Wang PJ, Yu JX, Jin S, Chen S, Yue C, Wang WL, Gao SL, Cao HL, Zheng YC, Gu MY, Chen XJ, Sun Y, Guo YQ, Yang JF, Zhang XT, Ye NX (2021) Genetic basis of high aroma and stress tolerance in the oolong tea cultivar genome. Horticulture Research 8 (1):107

Wang YP, Tang HB, DeBarry JD, Tan X, Li JP, Wang XY, Lee TH, Jin HZ, Marler B, Guo H, Kissinger JC, Paterson AH (2012) MCScanX: a toolkit for detection and evolutionary analysis of gene synteny and collinearity. Nucleic Acids Research 40 (7):e49

Xu Z, Wang H (2007) LTR_FINDER: an efficient tool for the prediction of full-length LTR retrotransposons. Nucleic Acids Research 35 (suppl_2):W265-W268

Yang J, Meyers KJ, Van Der Heide J, Liu RH (2004) Varietal differences in phenolic content and antioxidant and antiproliferative activities of onions. Journal of Agricultural and Food Chemistry 52 (22):6787-6793

Zhang JS, Zhang XT, Tang HB, Zhang Q, Hua XT, Ma XK, Zhu F, Jones T, Zhu XG, Bowers J, Wai CM, Zheng CF, Shil Y, Chen S, Xu XM, Yue JJ, Nelsons DR, Huang LX, Li Z, Xu HM, Zhou D, Wang YJ, Hu WC, Lin JS, Deng YJ, Pandey N, Mancini M, Zerpa D, Nguyen JK, Wang LM, Yu L, Xin YH, Ge LF, Arro J, Han JO, Chakrabarty S, Pushko M, Zhang WP, Ma YH, Ma PP, Lv MJ, Chen FM, Zheng GY, Xu JS, Yang ZH, Deng F, Chen XQ, Liao ZY, Zhang XX, Lin ZC, Lin H, Yan HS, Kuang Z, Zhong WM, Liang PP, Wang GF, Yuan Y, Shi JX, Hou JX, Lin JX, Jin JJ, Cao PJ, Shen QC, Jiang Q, Zhou P, Ma YY, Zhang XD, Xu RR, Liu J, Zhou YM, Jia HF, Ma Q, Qi R, Zhang ZL, Fang JP, Fang HK, Song JJ, Wang MJ, Dong GR, Wang G, Chen Z, Ma T, Liu H, Dhungana SR, Huss SE, Yang XP, Sharma A, Trujillo JH, Martinez MC, Hudson M, Riascos JJ, Schuler M, Chen LQ, Braun DM, Li L, Yu QY, Wang JP, Wang K, Schatz MC, Heckerman D, Van Sluys MA, Souza GM, Moore PH, Sankoff D, VanBuren R, Paterson AH, Nagai C, Ming R (2018) Allele-defined genome of the autopolyploid sugarcane *Saccharum spontaneum* L. Nature Genetics 50 (11):1565–1573

Zhang T, Liu G, Zhao H, Braz GT, Jiang J (2021) Chorus2: design of genome‐scale oligonucleotide‐based probes for fluorescence in situ hybridization. Plant Biotechnology Journal 19 (10):1967-1978
